# Assessment of Heme and Non-Heme Iron Intake and Its Dietary Sources among Adults in Armenia

**DOI:** 10.3390/nu15071643

**Published:** 2023-03-28

**Authors:** Davit Pipoyan, Seda Stepanyan, Meline Beglaryan, Alberto Mantovani

**Affiliations:** 1 Center for Ecological-Noosphere Studies, NAS RA, Yerevan 0025, Armenia; seda.stepanyan@cens.am (S.S.); meline.beglaryan@cens.am (M.B.); 2 National Institute of Health, 00161 Rome, Italy; alberto.mantovani@iss.it

**Keywords:** dietary iron intake, heme and non-heme iron, animal and plant products, sources of iron

## Abstract

Adequate dietary iron (Fe) intake is crucial for preventing Fe-deficient anemia, a recognized global public health concern which is important in Armenia. This study aimed to analyze the intake of Fe, both heme (from animal tissues) and non-heme (more prevalent, but less efficiently absorbed), as well as the Fe dietary sources, among adults in a representative national sample in Armenia. The study was conducted on 1400 individuals aged 18–80 and above, who were enrolled from all regions of Armenia. The Fe intake was assessed through a 24 h dietary recall survey, while Fe occurrence was determined through atomic absorption spectrophotometry (AAS). The results showed a high proportion of adults with a Fe intake lower than the average requirements set by EFSA (65%, 80% and 85% of males, total females and females at fertile age, respectively). Main Fe sources were bread, fruits and vegetables; heme Fe accounted only for <5% of total Fe intake. Compared to males, females had a lower intake of all forms of Fe (*p* < 0.05). Significant differences were observed in the intake of different forms of Fe between regions (*p* < 0.05), while the age-group 36–55 years had higher intakes of total Fe. Our data call for comprehensive nutritional security strategies in order to reduce iron deficiency in Armenia, that represents a public health concern.

## 1. Introduction

Iron (Fe) is the fourth most common element in the Earth’s crust. It is essential for oxygen transport, electron transfer, oxidase activities and energy metabolism [1]. When the supply of iron is insufficient, iron deficiency may develop. Iron deficiency is one of the most common nutritional disorders in the world and is estimated to be responsible for 50% of all anemia, globally. According to the World Health Organization (WHO) anemia is a significant global health problem that affects both developing and developed countries [2]. Iron deficiency anemia (IDA) has serious consequences for human health such as poor physical performance, impairment of RNA synthesis and neurotransmitter metabolism [1,2].

Anemia is a long-time public health concern in Armenia, as well as in other Countries of the composite Caucasus region [3,4]. According to the 2015-2016 Armenia Demographic and Health Survey (ADHS), anemia rates have decreased compared to 2000 data (from 25% to 13% among women and from 37% to 16% among children) [5]. Based on WHO global health observatory data, 17.3% of women of reproductive age (18–49 years old) are affected by anemia [6]. Recent data analyzed by World Food Programme (WFP) shows that food-insecure households have poorer diets in terms of both quantity and quality, and they have lower consumption of iron-rich foods, particularly meat and fish products. Regional differences in nutritional security may be marked in Caucasus countries: overall, north-western Armenia has the highest levels of food insecurity and the highest prevalence of malnutrition among children under 5. Among Armenia provinces, in 2015 the prevalence of anemia among both children and women ranged from 7% in Armavir to 39% in Gegharkunik [7,8]. In a neighboring Caucasus country, Georgia, the prevalence of anemia was also estimated to be high among children (36% were anemic, 74% of which with IDA) and pregnant women (21% were anemic, 57% of which with IDA) [9]. In another neighboring Caucasus country, Turkey, iron deficiency is the leading (90%) cause of anemia and is widespread especially among women [10]. While many foods, from meat through to legumes, are dietary sources of Fe, a robust assessment of Fe intake should distinguish between the heme iron, from animal tissues, and the non-heme iron which represent the vast majority (90%) of total dietary Fe but is less efficiently absorbed (about 10% vs. 25% of heme iron). It is important to note that despite some diets containing the recommended amount of iron, the bioavailability of non-heme iron may be limited due to the presence of other dietary components that can inhibit (e.g., phytate, polyphenols, calcium) or enhance (e.g., vitamin C) its absorption [1]. 

Based on ADHS data, the Ministry of Health (MOH) of Armenia advocates for a flour fortification program proposed to the parliament to prevent and control anemia in Armenia [11]. However, this ADHS used only hemoglobin to assess anemia, which is not a stand-alone indicator to plan iron fortification intervention, since hemoglobin measurement cannot alone determine the cause of the anemia [2]. When iron interventions are monitored by specific biomarkers of iron status, there is ample evidence that regular consumption of iron-fortified foods markedly improves iron status [12,13]. However, anemia prevalence alone cannot be used to monitor iron fortification interventions because the additional iron will only impact the proportion of anemia resulting from iron deficiency and not anemia resulting from other causes [12]. Besides, interventions to increase iron intake should consider also the risk of Fe excess in subjects sensitive to Fe overload, such as persons homozygous for haemochromatosis [1]. The prevalence of individuals susceptible to iron overload may be close to 2% in a given population [14]; however, limited data suggest that homozygosity for hemochromatosis has low prevalence in Armenia [15]. Considering the consequences of iron deficiency anemia, as well as the uncertainties of the iron fortification program, it is necessary to assess the iron intake of the Armenian population by an appropriate set of indicators. Hence, the aim of this study was to analyze iron intake derived from animal and plant products, to estimate heme, and non-heme iron intake, and to characterize iron dietary sources in the adult population across Armenia by means of a 24 h recall survey.

## 2. Materials and Methods

### 2.1. Food Sampling and Analysis

Food selection and sampling procedures were part of the total diet study (TDS). Food commodities that were both highly consumed as well as recognized sources of iron were considered for the current research. The main sources of iron in the analyzed group are bread and flour-based products, milk and milk products, meat and meat products, fish, egg, fruits, vegetables, potato, coffee, and water. These food groups (with 28 related sub-groups in total) represent the main products in Armenian diet [16] (Supplementary Materials, Table S1) and they include most of the foods that are recognized as main sources of iron by EFSA [1]. Each studied food item was a composite sample formed through pooling at least 8 individual samples (i.e., sub-samples) (Supplementary Materials, Table S1). The sample preparation, digestion processes and analysis are described by Pipoyan et al. [16].

### 2.2. Food Consumption Data Collection

The dietary survey included a 24 h recall used to obtain data on food consumption by Armenia’s adult population (18–80 years old and above). This method is the most efficient one for diet investigation [17]. The data collection period was from February to September 2021. The information was collected by well-trained interviewers via face-to-face and telephone interviews, using pre-designed forms. Overall, 1400 residents of Armenia were interviewed. This survey included all the regions of Armenia: Armavir, Ararat, Aragatsotn, Gegharkunik, Kotayk, Lori, Shirak, Syunik, Tavush, Vayots Dzor, and the capital city, Yerevan. 

To obtain a representative national sample of Armenian adult population, a stratified sampling was used. In particular, random sampling quotas were designed for: regions of Armenia, gender as well as age group proportions. Namely age groups were 18–35 (*n* = 505), 36–55 (*n* = 478), 56–79 (*n* = 366), 80 years old and above (*n* = 51). In case of Yerevan, to obtain more accurate and representative data, survey participants were chosen from 12 administrative districts with equal access.

### 2.3. Estimation of Iron Intake

Daily iron intake was calculated according to the following formula:

EDI = IR × C(1)

where EDI is the estimated daily intake of iron (mg/day). IR is the daily ingestion rate of each food product (kg/day) and C is the iron content (mg/kg and mg/L). 

The iron intake was calculated for specific food product groups: bread and flour-based products, milk and milk products, meat and meat products, fish, egg, fruits and vegetables, potato, coffee, and water. The total daily iron intake was obtained as the sum of the values of iron intake from all groups of products. Afterwards, the iron intake was calculated for specific forms of iron: animal iron and plant iron. Heme and non-heme iron intakes were estimated considering that heme iron is attributed to 40% of iron derived from animal products, while non-heme iron is attributed to 60% of iron derived from animal products and 100% of iron derived from plant products [18].

The total iron intake, estimated heme and non-heme iron intake, and iron intake from each food product groups were compared between different regions, gender, and age groups. The percentage (%) of individuals that consumed foods rich in heme, non-heme iron, as well as iron of animal and plant origin during the last 24 h has been calculated using the following formula [19]:

(2) $$\frac{Number\;of\;individuals\;that\;consumed\;heme/none\;heme/animal/plant\;iron\;rich\;foods}{Total\;number\;of\;respondents}\times 100$$

The calculated values for the total iron intake were compared with the Recommended Dietary Allowance (RDA) level set by the Institute of Medicine (Institute of Medicine, 2001), Population Reference Intake (PRI) and Average Requirement (AR) set by the European Food Safety Authority (EFSA) [1] (Table 1). 

### 2.4. Statistical Analysis

To check for the homogeneity of food consumption data, normality tests were conducted. Based on the tests, the significance value of the Shapiro-Wilk test was below 0.05, indicating that the consumption data significantly deviates from a normal distribution. Therefore, due to a nonparametric distribution, Mann-Whitney U test and Kruskal-Wallis analysis of variance (ANOVA) were used to compare different types of iron intake between sub-groups (region, gender and age), with a *p*-value of less than or equal to 0.05 as a level of significance. A Mann-Whitney U test was used to compare iron intake between two groups of interest: males and females. In case of three or more groups (such as region and age), a Kruskal-Wallis one-Way ANOVA was used since the consumption data is skewed. All the statistical analysis was completed via IBM SPSS Software (SPSS Inc., Chicago, IL, USA, version 28).

## 3. Results and Discussion

Intake of iron from different food sources and in various forms among a national sample of Armenian male and female adults is presented in Table 2. As it can be seen, female respondents are characterized by a significantly lower (*p* < 0.05) total iron intake—including heme iron, non-heme iron, animal iron, and plant iron—than the male respondents. Regarding the iron intake from various sources, female respondents receive lower amounts from bread products, milk products, meat products, eggs, potatoes, fruits, vegetables, black coffee and tap water than male respondents, except for a slightly, not statistically significant, higher amount from fish, (*p* = 0.23).

Intake of iron from various sources and in various forms among a national sample of Armenian adults separated by all the regions is presented in Table 3. The data show a significant difference between iron intakes among Armenian regions. Ararat is characterized by the highest mean total iron intake (19.57 mg), while Yerevan has the lowest value (14.70 mg) (*p* < 0.05). Heme and animal iron intakes are the highest in the Gegharkunik region and the lowest in theArmavir region (*p* < 0.05). The Gegharkunik region was characterized also by the higher iron intake from meat products (*p* < 0.05). Intake of iron from various sources and in various forms among different age groups is presented in Table 4. Overall, people aged from 36 to 55 tended to have a relatively higher intake of iron than the other age groups; the difference was significant (*p* < 0.05) in regard to total Fe and plant-derived Fe. Conversely, the age group aged 80 and above have the lowest intake of all forms of iron. 

The share of respondents, characterized by a recommended Intake and intake lower than recommended, was compared between different regions, gender, and age groups (Table 5). Overall, there is a very high percentage of respondents who are characterized by iron intake lower than the recommended levels of RDA, PRI, and AR. The percentages are higher when comparing with RDA and PRI, since these figures are much higher than AR. At least 70% and at least 88% of respondents have an iron intake lower than RDA and PRI, respectively. Approximately 65% of males and 80% of females are characterized by inadequate intake of iron (lower than AR). Almost 85% of adult females of reproductive age (18–49 years) [20] have an iron intake lower than AR. Regarding regions, almost 87% of the population of Yerevan has an iron intake lower than AR. The two regions with the lowest share of respondents who have an iron intake lower than AR are Gegharkunik (51%) and Lori (50%); also, in these regions, half of respondents have an inadequate intake.

In the national sample of Armenian adults, plant iron intake accounts for 89% of total iron intake, while animal iron accounts for only 11%. Accordingly, non-heme iron makes almost 95% of total iron intake, and heme iron makes only 5% (Table 2). When looking at the share of iron from various food sources, the primary sources of iron for all the respondents are bread products (almost 33%).

The share of respondents with Intake lower than recommended was compared between different regions, gender, and age groups (Table 5). Overall, there is a very high percentage of respondents who are characterized by iron intake lower than the recommended levels of RDA, PRI, and AR. The percentages are higher when comparing with RDA and PRI, since these figures are much higher than AR. At least 70% and at least 88% of respondents have an iron intake lower than RDA and PRI, respectively. Approximately 65% of males and 80% of females are characterized by inadequate intake of iron (lower than AR). Almost 85% of adult females of reproductive age (18–49 years) [20] have an iron intake lower than AR. Regarding regions, almost 87% of the population of Yerevan has an iron intake lower than AR. The two regions with the lowest share of respondents who have an iron intake lower than AR are Gegharkunik (51%) and Lori (50%); also, in these regions, half of respondents have an inadequate intake.

In our national sample of Armenian adults, plant iron intake accounts for 89% of total iron intake, while animal iron accounts for only 11%. Accordingly, non-heme iron makes up almost 95% of total iron intake, and heme iron makes only 5% (Table 2). When looking at the share of iron from various food sources, the primary sources of iron for all the respondents are bread products (almost 33%), which is followed by fruits (averaging approximately 28%), vegetables (approximately 15%), then at a distance by potato (approximately 7%) and milk products (approximately 5.5%). These are food groups that contain relatively low content of iron [1]. Conversely, a high amount of iron is present in meat and meat products [1]: in our study, these foods account for only 3% to 4% of total iron intake.

To derive the high-risk groups of iron deficiency or overload, the iron intake is also calculated considering two boundaries of consumption distribution in the studied population: 5th and 95th percentiles by less than 4.7 mg and 39.11 mg of total iron intake, respectively. Accordingly, the estimated heme and non-heme iron intakes range from 0.23 to 1.64 mg and 4.47 to 37.47 mg, respectively. Our data on main dietary contributors and the predominance of non-heme iron are consistent with previous research suggesting that the primary sources of iron for most of the Armenian population are bread and potato [21,22]. 

Meat products contain heme iron, which is both more bioavailable than non-heme iron and enhances the absorption of non-heme iron present in the same meal [2]. In the presence of a relatively low intake of total iron, a high proportion of iron from plant-based products and a limited intake of iron of animal origin can increase the risk of IDA due to the poor absorption of iron from plant-based diets and meals [23,24]. Hence, the current picture indicates a widespread concern for IDA in the adult Armenian population due to low levels of total iron with predominance of non-heme iron. The reasons for such a widespread iron-deficient diet are diverse, including food accessibility constraints, such as insufficient resources to purchase iron-rich products, and educational constraints, such as lack of proper nutritional education and knowledge about sources of iron [21,22]. According to the FAO, factors contributing to anemia among Armenian population could be the presence of inhibitors of iron absorption in cereals (e.g., phytates and tannins) [25], the rare consumption of meat due to its high cost, the seasonal consumption of fruits and vegetables (containing enhancers of iron absorption), and the widespread and frequent consumption of tea and coffee (containing strong inhibitors of iron absorption) [26]. Hence, a further step would be to incorporate the bioactive substances modifying—either enhancing or reducing—absorption into dietary intake assessment. The combination of social and nutritional factors may not only relate to iron deficiency; previous studies highlighted that in Armenia, there are several nutrient deficiencies, such as lack of vitamin A [22]. Overall, our data support that equitable access to nutritional security in Armenia is still a goal to be achieved.

To address iron-deficient anemia, the WHO developed nutrition-sensitive and nutrition-specific interventions. The main nutrition-sensitive interventions address the underlying and basic causes of anemia, such as diseases (malaria, intestinal helminths), water, sanitation, hygiene, lack of education, poverty, etc. [2]. In Armenia, malaria has been eradicated at the beginning of the 1960s, and no single case of indigenous transmission of malaria was reported until now [27]. Meanwhile, intestinal helminths continue to be a public health problem in the country [28,29]. While the majority of the population has an university education level, the overall awareness regarding nutrition and dietary recommendations is still low [3,30]. The poverty rate is high and food expenditures represent more than half of the household’s income [22].

The main nutrition-specific interventions include dietary diversification, iron fortification and supplementation [2]. Dietary diversification is recommended mostly in low- and middle-income countries where diets are often monotonous and poor in micronutrients. The advantage of this approach is that it simultaneously combats multiple micronutrient deficiencies and may therefore be very useful for a country such as Armenia, where there are several nutrient deficiencies [22,31]. However, this approach requires a long-term practice, and it presents issues related to affordability; this is a major concern for Armenia, where the poverty rate is high.

Mass fortification of iron is recommended when most of the population is exposed to a public health risk of being or becoming iron-deficient [32]. Flour fortification programs with iron were introduced in the 1940s to target widespread anemia and are now mandatory in 81 countries [12]. However, in several low- and middle-income countries, this practice revealed controversial outcomes. While many studies provide evidence of the effectiveness of this approach [2,33,34], some others indicate that it has resulted in only modest decreases in anemia prevalence since in such countries, infections, inflammation, diseases, and other nutritional deficiencies are concurrent and often more important causes of anemia than iron deficiency [12]. However, our data show that this may not be the case for Armenia. In addition, the bioavailability of iron should be considered when deciding fortification levels, the high presence of antinutritional factors might also reduce the favorable impact of fortification programs. Moreover, the high consumption of iron-fortified wheat or maize flours may increase the risk of becoming overweight [35]; thus, advocacy of iron fortification should not become a strenuous promotion of carbohydrate-rich diets. In addition, the fraction of people with hemochromatosis deserves attention, as iron overload can cause serious chronic illness.

Supplementation of iron is recommended in iron-deficient settings, particularly among vulnerable groups of children and pregnant women. This approach is effective and rather inexpensive. However, high doses of iron supplements may cause adverse health effects, such as stomach pain, nausea, or constipation [36]. While such effects occur at supplemental intakes of at least 60 mg [1], it may be difficult to control the intake of supplements, especially when widely advertised.

Overall, the different approaches to reduce iron deficiency may present shortcomings and even risks, and they are therefore liable to a context-specific, comparative benefit-to-risk assessment [37].

## 4. Conclusions

This is the first-ever investigation to determine total, heme and non-heme iron intake using a 24 h recall survey in a country of the Caucasus region, where iron deficiency is a public health concern. The findings of this study showed that a majority of the population has an inadequate iron intake and may be at risk of IDA. Additionally, the contribution of meat products and heme iron to total iron intake was found to be relatively low; hence, the limited bioavailability of the dietary iron consumed can worsen the impact of the overall poor intake. To address this issue, a combination of nutrition-sensitive and nutrition-specific interventions is recommended. Possible strategies include mass fortification of flour, increased consumption of meat products and fish, reduction in poverty, and improved nutrition literacy. However, it is important to implement these interventions comprehensively and based on food-based guidelines while also considering potential drawbacks and adverse effects. At present, the only strategic initiative planned in Armenia is the fortification of foods with micronutrients [11]; however, this plan has yet to be put into action. It is suggested that a comprehensive, country-specific strategy be developed that integrates fortification with additional approaches such as iron supplementation, dietary diversification, and enhancement of nutrition literacy.

## 5. Study Limitations

A limitation of the study is the use of a 1-day 24 h recall. This method lacks precision when estimating within-subject variation in intake, since it does not represent a habitual diet at an individual level. For calculating specific nutrient adequacy, it is suggested to use methods that capture the consumption frequency and amounts of all foods contributing the dietary component of interest. The most recommended one is the multiple-pass 24 h diet recall taken on several days [38,39]; the use of this approach might have improved the accuracy of estimates by incorporating the contribution of less frequently consumed foods. However, our approach has sufficient statistical power to capture the main qualitative and quantitative features of a major and widespread public health issue such as iron deficiency in Armenia.

## Figures and Tables

**Table 1:** Dietary reference intake recommendations for iron.

Reference Values	Males and Postmenopausal Females	Premenopausal Females
Recommended Dietary Allowance (RDA)	8 mg/day	18 mg/day
Population Reference Intake (PRI)	11 mg/day	16 mg/day
Average Requirement (AR)	6 mg/day	7 mg/day

**Table 2:** Comparison of intake of various forms of iron along with that of iron intake from various sources in the national sample of Armenian adults among male and female respondents.

Intake of Iron (mg/day)		Male Respondents (*n* = 666)		Female Respondents (*n* = 734)		*p*-Value *
		Intake (%)	Mean ± SD	Intake (%)	Mean ± SD	
Intake of various forms of iron	Total iron	100	17.81 ± 0.91	100	15.58 ± 0.77	<0.05
	Heme iron	4.3	0.77 ± 0.05	4.0	0.69 ± 0.05	<0.05
	Non-heme iron	95.7	17.04 ± 1.16	96.0	14.88 ± 0.98	<0.05
	Animal iron	10.8	1.93 ± 0.13	11.0	1.73 ± 0.12	<0.05
	Plant iron	89.2	15.88 ± 1.08	89.0	13.84 ± 0.91	<0.05
Intake of iron from various sources	Bread and flour-based products	33.5	5.97 ± 1.09	32.6	5.07 ± 0.90	<0.05
	Milk and milk products	5.3	0.95 ± 0.09	5.6	0.88 ± 0.08	<0.05
	Fat and oil products	0.1	0.01 ± 0.00	0.1	0.01 ± 0.00	0.064
	Meat and meat products	3.8	0.68 ± 0.16	3.4	0.53 ± 0.13	<0.05
	Fish	1.6	0.29 ± 0.00	2.0	0.32 ± 0.00	0.233
	Eggs	0.0	0.01 ± 0.00	0.0	0.01 ± 0.00	<0.05
	Fruits	26.5	4.71 ± 1.85	26.1	4.06 ± 1.51	0.295
	Vegetables	21.5	3.83 ± 0.41	21.8	3.40 ± 0.29	<0.05
	Potato	6.4	1.13 ± 0.00	7.2	1.11 ± 0.00	0.743
	Black coffee	0.6	0.11 ± 0.00	0.6	0.10 ± 0.00	0.453
	Tap water	0.7	0.12 ± 0.00	0.6	0.09 ± 0.00	<0.05

Note: * Compared with independent Samples Mann-Whitney U test due to nonparametric distribution (verified using Shapiro-Wilk test; *p* ≤ 0.05). The significance level is 0.05.

**Table 3:** Comparison of intake of various forms of iron along with that of iron intake from various sources in the national sample of Armenian adults from all the regions.

Intake of Iron (mg/day)		Yerevan (*n* = 578)	Araga-tsotn (*n* = 52)	Ararat (*n* = 104)	Arma- vir (*n* = 111)	Geghar- kunik (*n* = 96)	Lori (*n* = 97)	Kotayk (*n* = 116)	Shirak (*n* = 93)	Syunik (*n* = 68)	Vayots Dzor (*n* = 34)	Tavush (*n* = 51)	*p*-Value *
Intake of various forms of iron	Total iron	14.70	16.66	19.57	17.10	17.55	16.30	16.62	16.47	18.04	17.42	15.65	<0.05
	Heme iron	0.69	0.55	0.80	0.51	0.96	0.56	0.62	0.83	0.66	0.57	0.61	<0.05
	Non-heme iron	14.01	16.12	18.77	16.59	16.59	15.74	16.00	15.64	17.39	16.85	15.04	<0.05
	Animal iron	1.72	1.37	1.99	1.27	2.40	1.39	1.56	2.08	1.64	1.42	1.52	<0.05
	Plant iron	12.98	15.29	17.57	15.83	15.15	14.91	15.06	14.39	16.40	16.00	14.13	<0.05
Intake of iron from various sources	Bread and flour-based products	5.17	5.66	5.31	5.53	6.03	4.95	6.60	5.52	5.82	6.11	5.04	<0.05
	Milk and milk products	0.93	0.68	0.84	0.63	0.90	0.74	0.94	1.16	0.87	0.78	0.71	<0.05
	Fat and oil products	0.01	0.01	0.00	0.00	0.01	0.01	0.01	0.01	0.01	0.01	0.00	0.14
	Meat and meat products	0.52	0.34	0.88	0.40	1.13	0.64	0.60	0.54	0.76	0.27	0.25	<0.05
	Fish	0.26	0.35	0.26	0.23	0.35	0.00	0.00	0.37	0.00	0.36	0.56	0.48
	Eggs	0.01	0.00	0.01	0.00	0.01	0.01	0.01	0.01	0.01	0.01	0.01	<0.05
	Fruits	3.52	4.13	6.68	5.65	4.65	4.98	2.87	3.98	4.64	4.46	4.44	<0.05
	Vegetables	3.00	4.35	4.16	3.44	2.92	3.60	4.22	3.55	4.51	4.08	3.44	<0.05
	Potato	1.10	0.94	1.16	1.00	1.30	1.17	1.17	1.10	1.20	1.13	1.01	0.59
	Black coffee	0.10	0.11	0.10	0.10	0.12	0.10	0.12	0.10	0.13	0.11	0.08	<0.05
	Tap water	0.08	0.10	0.16	0.11	0.13	0.12	0.08	0.13	0.11	0.11	0.12	<0.05

Note: * Compared with independent Samples Kruskal-Wallis test due to nonparametric distribution (verified using Shapiro-Wilk test; *p* ≤ 0.05). The significance level is 0.05.

**Table 4:** Comparison of intake of various forms of iron along with that of iron intake from various sources in the national sample of Armenian adults among different age groups.

Intake of Iron (mg/day)		18–35 Years Old (*n* = 505)	36–55 Years Old (*n* = 478)	56–79 Years Old (*n* = 366)	80 Years Old and Above (*n* = 51)	*p*-Value *
Intake of various forms of iron	Total iron	16.38	17.08	16.53	15.67	<0.05
	Heme iron	0.75	0.78	0.65	0.48	0.253
	Non-heme iron	15.62	16.29	15.87	15.18	0.224
	Animal iron	1.88	1.96	1.63	1.21	0.253
	Plant iron	14.49	15.11	14.90	14.46	<0.05
Intake of iron from various sources	Bread and flour-based products	5.38	5.61	5.57	5.42	0.273
	Milk and milk products	1.00	0.9	0.87	0.80	0.321
	Fat and oil products	0.01	0.01	0.01	0.00	0.07
	Meat and meat products	0.57	0.70	0.51	0.12	0.615
	Fish	0.30	0.35	0.23	0.28	0.31
	Eggs	0.01	0.01	0.01	0.01	0.331
	Fruits	4.42	4.37	4.20	4.72	0.096
	Vegetables	3.39	3.82	3.73	2.95	0.255
	Potato	1.11	1.10	1.18	1.16	<0.05
	Black coffee	0.10	0.11	0.10	0.10	<0.05
	Tap water	0.10	0.10	0.16	0.11	<0.05

Note: * Compared with independent Samples Kruskal-Wallis test due to nonparametric distribution (verified using Shapiro-Wilk test; *p* ≤ 0.05). The significance level is 0.05.

**Table 5:** Share of individuals characterized by adequate or inadequate iron intake.

Variable	Sub-Groups	<RDA *	<PRI **	<AR ***	>RDA *	>PRI **	>AR ***
Gender	Males	539 (80.9%)	623 (93.5%)	432 (64.8%)	127 (19.1%)	43 (5.5%)	234 (35.2%)
	Females	689 (93.8%)	717 (97.6%)	582 (79.3%)	45 (6.2%)	17 (2.4%)	152 (20.7%)
Age Groups	18–35	465 (92.1%)	488 (96.6%)	389 (77.0%)	40 (7.9%)	17 (3.4%)	116 (33.0%)
	36–55	427 (89.3%)	457 (95.6%)	346 (72.3%)	51 (10.7%)	21 (4.4%)	132 (27.6%)
	56–79	331 (90.4%)	346 (94.5%)	244 (66.6%)	35 (9.6%)	20 (5.5%)	122 (33.3)
	80+	47 (92.1%)	49 (96.1%)	37 (72.5%)	4 (7.9%)	2 (3.9%)	14 (27.5%)
Reproductive Age	18–49 Years Old Males	353 (83.1%)	396 (93.1%)	287 (67.5%)	72 (16.9%)	29 (6.9%)	138 (32.5%)
	18–49 Years Old Females	423 (99.5%)	422 (99.2%)	359 (84.4%)	2 (0.5%)	3 (0.8%)	66 (15.6%)
Region	Yerevan	547 (94.6%)	572 (98.9%)	501 (86.6%)	31 (5.4%)	6 (1.1%)	77 (13.4%)
	Aragatsotn	45 (86.5%)	50 (96.1%)	36 (69.2%)	7 (13.5%)	2 (3.9%)	16 (30.8%)
	Ararat	86 (82.6%)	98 (94.2%)	69 (66.3%)	18 (17.3%)	6 (5.8%)	35 (33.7%)
	Armavir	101 (90.9%)	108 (97.2%)	87 (78.3%)	10 (9.1%)	3 (2.8%)	24 (21.7%)
	Gegharkunik	78 (81.25%)	87 (91.0%)	49 (51.0%)	18 (18.75%)	9 (9.0%)	47 (49.0%)
	Lori	71 (73.2%)	89 (90.0%)	49 (50.5%)	26 (26.8%)	8 (10.0%)	48 (49.5%)
	Kotayk	94 (81.0%)	108 (93.1%)	70 (60.3%)	22 (19%)	8 (6.9%)	46 (39.7%)
	Shirak	79 (84.9%)	86 (92.4%)	63 (67.7%)	14 (15.1%)	7 (7.6%)	30 (32.3%)
	Syunik	51 (75%)	62 (91.1%)	38 (55.8%)	17 (25%)	6 (8.9%)	30 (44.2%)
	Vayots Dzor	30 (88.2%)	30 (88.2%)	22 (64.7%)	4 (11.8%)	4 (11.8%)	12 (35.3%)
	Tavush	46 (90.2%)	50 (98.0%)	30 (58.8%)	5 (9.8%)	1 (2.0%)	21 (41.2%)

Note: * RDA (Recommended Dietary Allowance; For males above 19 and females aged 51 and above—8 mg/day, females aged 19–50—18 mg/day). ** PRI (Population Reference Intake; for males and postmenopausal females—11 mg/day, premenopausal females—16 mg/day). *** AR (Average Requirement; for males and postmenopausal females—6 mg/day, premenopausal females—7 mg/day).

## Data Availability

Data are contained within the article.

## Supplementary Materials

The following supporting information can be downloaded at: https://www.mdpi.com/article/10.3390/nu15071643/s1, Table S1: Investigated food products. Reference [16] is cited in the supplementary materials.
