# Supplementary material for: Assessment of Heme and Non-Heme Iron Intake and Its Dietary Sources among Adults in Armenia
Source: Nutrients. 2023 Mar 28;15(7):1643. doi: 10.3390/nu15071643 (PMC10097195; doi:10.3390/nu15071643)
Supplement: Supplementary file 1 [file nutrients-15-01643-s001.zip › nutrients-2313171-supplementary.pdf]

Table S1. Investigated food products.

Food Groups	Composite Food Products ("As Consumed" Preparation)	N of Individual Samples (Sub-Samples)
Bread and flour-based products	Bread	16
	Lavash	16
	Rice (boiled in tap water with salt and spices, as well as prepared as a pilaf)	8
	Buckwheat (boiled in tap water with salt, then vegetable oil was added)	8
	Emmer and groat (boiled in tap water with salt, then vegetable oil was added)	8
	Macaroni and vermicelli (boiled in tap water with salt, then vegetable oil was added)	8
	Waffles and cookies	10
Milk and milk products	Pasteurized milk	8
	Yogurt	8
	Sour cream	8
	Matsun	8
	Cheese	8
	Curd/cottage cheese	8
Meat and meat products	Beef and veal (boiled, pan-fried, or prepared as a barbeque)	8
	Pork (grilled or prepared as a barbeque)	8
	Chicken meat (boiled, pan-fried, grilled, or prepared as a barbeque)	8
	Sausage products	8
	Pelmeni and khinkali	8
Fish	Fish meat (boiled or pan-fried)	8
Egg	Chicken egg (boiled or prepared as an omelet)	8
Fat and oil products	Butter, margarine, and vegetable oils	10
Fruits	Fruits	14
Vegetables	Tomato, pepper, eggplant, zucchini, and green bean (grilled/fried in vegetable oil or smoked)	8
Watermelon and melon	Watermelon and melon (washed and pilled)	8
Tomato	Fresh tomatoes	8
Cucumber	Cucumber (washed and pilled)	8
Potato	Potatoes (boiled, or fried in vegetable oil)	8
Coffee	Black coffee (prepared with tap water, without sugar)	10
Water	Tap water	12

Note [1]*:* [1] Lavash is a thin flatbread, commonly baked by women in countries across the Caucasus and Middle East.
[2] Emmer and groat are used in Armenia to make traditional pilav (acharov plav, harisa) and soup (spas).
[3] Matsun is a fermented milk product (prepared from buffalo, goat, or cow milk, or their combinations) similar to yogurt. It has high consumption in West Asia, especially in Armenia.
[4] Pelmeni is a meat dumpling, common in the cuisine of Russia and post-Soviet countries. Khinkali is an herb- and meat-filled dumpling, commonly made in Georgia and other Caucasian countries, including Armenia.

Reference

1. Pipoyan, D.; Stepanyan, S.; Beglaryan, M.; Mantovani, A. Risk Characterization of the Armenian Population to Nickel: Application of Deterministic and Probabilistic Approaches to a Total Diet Study in Yerevan City. *Biol. Trace Elem. Res.* **2022**, *ahead of print*. https://doi.org/10.1007/s12011-022-03371-8.
